# Symptoms, complications and management of long COVID: a review

**DOI:** 10.1177/01410768211032850

**Published:** 2021-07-15

**Authors:** Olalekan Lee Aiyegbusi, Sarah E Hughes, Grace Turner, Samantha Cruz Rivera, Christel McMullan, Joht Singh Chandan, Shamil Haroon, Gary Price, Elin Haf Davies, Krishnarajah Nirantharakumar, Elizabeth Sapey, Melanie J Calvert, on behalf of the TLC Study Group

**Affiliations:** 1 Institute of Applied Health Research, 1724 University of Birmingham, Birmingham, UK; 2 Centre for Patient Reported Outcomes Research, Institute of Applied Health Research, 1724 University of Birmingham, Birmingham, UK; 3 National Institute for Health Research (NIHR) Applied Research Centre West Midlands, Birmingham, UK; 4 Birmingham Health Partners Centre for Regulatory Science and Innovation, 1724 University of Birmingham, Birmingham, UK; 5 NIHR Birmingham Biomedical Research Centre, NIHR Surgical Reconstruction and Microbiology Research Centre, 1724 University of Birmingham, Birmingham, UK; 6 560911 Aparito Limited, Wrexham, UK; 7 Midlands Health Data Research UK, Birmingham, UK; 8 Birmingham Acute Care Research Group, Institute of Inflammation and Ageing, 1724 University of Birmingham, Birmingham, UK; 9 Acute Medicine, University Hospitals Birmingham NHS Foundation Trust, Edgbaston, Birmingham, UK; 10 Health Data Research UK, London, UK

**Keywords:** epidemiology, health service research, infectious diseases, public health, respiratory medicine, COVID-19, long COVID, post-COVID-19 syndrome, persistent COVID-19 symptoms

## Abstract

Globally, there are now over 160 million confirmed cases of COVID-19 and more than 3 million deaths. While the majority of infected individuals recover, a significant proportion continue to experience symptoms and complications after their acute illness. Patients with ‘long COVID’ experience a wide range of physical and mental/psychological symptoms. Pooled prevalence data showed the 10 most prevalent reported symptoms were fatigue, shortness of breath, muscle pain, joint pain, headache, cough, chest pain, altered smell, altered taste and diarrhoea. Other common symptoms were cognitive impairment, memory loss, anxiety and sleep disorders. Beyond symptoms and complications, people with long COVID often reported impaired quality of life, mental health and employment issues. These individuals may require multidisciplinary care involving the long-term monitoring of symptoms, to identify potential complications, physical rehabilitation, mental health and social services support. Resilient healthcare systems are needed to ensure efficient and effective responses to future health challenges.

## Introduction

**Figure 1.**
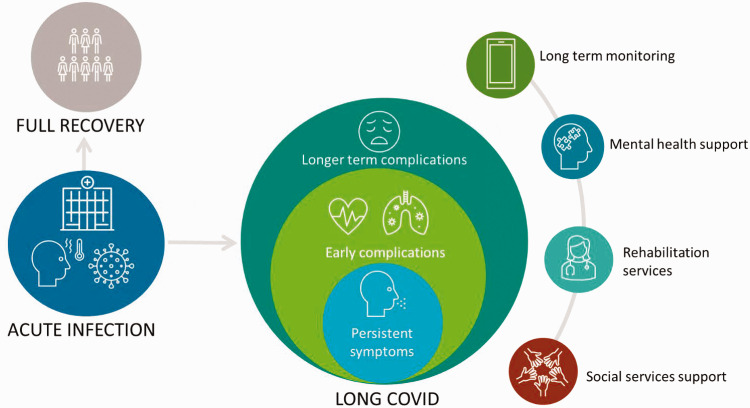
Depiction of the clinical course of long COVID.

Globally, there are now over 160 million confirmed cases of COVID-19 and more than 3 million deaths.^1^ The majority of people with COVID-19 experience mild-to-moderate illness, while approximately 10%–15% develop severe illness and 5% become critically ill.^2^ The average recovery time from COVID-19 is 2–3 weeks depending on symptom severity.^3–5^ However, 1 in 5 people, regardless of the severity of their acute infection, may exhibit symptoms for 5 weeks or more, while 1 in 10 may have symptoms lasting 12 weeks or more.^6^ There is yet to be a consensus on the appropriate definitions for situations where COVID-19 symptoms persist beyond the acute phase of infection. The patient-coined term ‘long COVID’ was proposed^7,8^ and there were calls for its full adoption in scientific literature.^9,10^ The UK’s current National Institute for Health and Care Excellence guideline states that long COVID encompasses ongoing symptomatic COVID-19 (where symptoms last for 4 to 12 weeks) and post-COVID-19 syndrome (when they persist beyond 12 weeks in the absence of an alternative diagnosis).^11^ Figure 1 depicts the potential clinical course of COVID-19. This review summarises the current evidence on symptom prevalence, complications and management of long COVID and highlights priority areas for research.

## Methods

We searched the Living Systematic Review database^12^ on 8 February 2021 for articles that described persistent COVID-19 symptoms. This database retrieves scientific publications related to COVID-19 from PubMed, EMBASE, MedRxiv and BioRxiv. We also conducted hand searching of reference lists of selected articles. The authors who performed the screening discussed and agreed on the included articles. Critical appraisal of the studies was conducted using the modified Newcastle Ottawa Scale.

## Eligibility criteria

### Inclusion

- Quantitative and qualitative studies of adults with ongoing symptomatic COVID-19 or post-COVID syndrome.
- Published since 1 January 2020.
- English language.

### Exclude

- Studies that focus only on acute COVID-19 (<4 weeks).
- Narrative reviews, commentaries, opinion pieces and letters that do not report primary findings.

### Symptomatology and lived experiences of long COVID

#### Search results

We retrieved 1809 entries from our database search and two independent researchers screened 1574 after removing duplicates. Full texts for 94 articles were examined and 24 were selected. Three more articles were identified through hand searching, bringing the total number of included articles to 27.

#### Long COVID symptoms

Patients with long COVID experience the persistence of a wide range of physical and mental/psychological symptoms. Table 1 shows the symptoms of long COVID described by 27 primary research articles we included in this review.^3,4,13–36^ According to National Institute for Health and Care Excellence classification, 19 articles described ongoing symptomatic COVID-19 (symptoms lasting 4–12 weeks)^3,4,13–19,21,22,24,25,27,28,31–33,36,37^ and 8 reported post-COVID-19 syndrome (symptoms persisting beyond 12 weeks).^18,20,23,26,27,30,34,36^ Three studies provided data for both categories.^18,27,36^ Box 1 summarises the critical appraisal of these studies.

**Table 1.** Signs and symptoms of long COVID.

	Symptoms
Cardiopulmonary	Fatigue^3,4,13,14,16,18,20–25,27,31,34–36,a^
	Shortness of breath (dyspnoea)^3,4,13–15,17–22,24,25,27,29,33,35,b^
	– Shortness of breath at rest^16^
	– Shortness of breath with exertion^16,17,30,34,36^
	Chest pain^3,4,13–15,18–21,23,25,30,35,36^
	Palpitations^13,15,16,18,21,23,36^
	Chest tightness^16,21^
	Wheezing^16,29^
Naso-oropharyngeal	Loss of smell (anosmia)^3,4,13–18,20,21,23,24,26,27,29,30,32,35,37,c^
	Dysgeusia (altered taste)^3,13,14,16–18,20,21,23,24,26,29,32,37,c^
	Sore throat^3,4,14,16,21–23,29^
	Cough^3,4,13,14,16–21,24,27,29,30,34,35^
	Tinnitus^13,18,25,36^
	Sputum production^14,24^
	Hoarse voice^4^/voice change^22^
	Aphonia^13^
	Rhinitis^14,21^/rhinorrhea^3,29^
	Sneezing^21^
	Chronic sinusitis/congestion^13,16^
	Ear pain^21,29^
	Hearing loss^26^
	Diarrhoea^3,4,14–16,18,21–24,27,29,35,d^
	Nausea^3,16,18,21^
	Loss of appetite^4,14,22,23^
	Abdominal pain^3,4,16,29,35^
	Weight loss^15,21^/anorexia^16,19^
	Vomiting^21,29^
	Gastritis^19^
Musculoskeletal	Joint pain (arthralgia)^13–16,18,19,21,23–25,27,29,30,35,e^
	Muscle pains (myalgia)^3,4,13–15,19,21,23,24,26,29,30,35,f^
Neuro-psychological	Memory loss (amnesia)^13,16,18,20,22,26,27,36^
	Difficulty thinking/inability to concentrate^18,20,22^/brain fog/cognitive impairment^3,36^/ disorientation^13,16,18,24,29^/delirium^4^
	Sleep disorders such as insomnia^13,16,18–20,23,28,30,35,36^
	Visual disturbances^13,21,25,27,29^
	Anxiety and depression^22^/anxiety^16,19,25,28,g^
	Depression^25,28^
	Mood change^26^
	Thoughts of self‐harm^22^/suicide^28^
	Neuralgia/neuropathy^13,26^/needle pains in arms and legs (paraesthesia)/tingling^18,36^
	Tremors^26^
	Seizures^29^
Miscellaneous	Fever/chills^3,4,13,15,16,18,21,23–25,29,35,36^
	Headache^3,4,13–16,18,21,23–27,29,34–36,f^
	Dizziness^18,36^/vertigo^14,16,21,23^
	Skin rash^18,23,29^/pruritus^13,16^/cutaneous signs^15,27^/red spots on feet^21^
	Significant hair loss^20,23^
	Red eyes^14^/eye irritation^24,29^
	Asthenia^30^/weakness^16,18^
	Unspecified pain^13^/body ache^16^
	Bladder incontinence^22^
	Hot flushes^21^
	Sweats^16^
	Sicca syndrome^14^
	Ulcer^24^

a Banda combined malaise and fatigue (International Classification of Diseases (ICD) 10 Code).

b Chopra combined shortness of breath/chest tightness/wheezing.

c Carvalho & Chopra & Moreno combined anosmia and ageusia.

d Huang recorded as diarrhoea or vomiting.

e Moreno combined muscle and joint pain.

f Carvalho combined myalgia, headache and/or asthenia.

g Anxiety/depression measured using EQ-5D-5 L.

**Box 1.** Critical appraisal of studies that reported the prevalence of long COVID symptoms.

The modified Newcastle Ottawa Scale was used to evaluate the quality of the included studies. The design and methodological issues identified are presented here. However, these issues have to be considered with caution given the fast and constantly evolving nature of the pandemic, which might make them unavoidable for researchers.
• Virtually all the primary articles considered for this review excluded individuals with issues such as delirium. This might have led to missing some of the neuropsychiatric complications of long COVID.
• While the studies reviewed reported the prevalence of ongoing symptoms, there was a general lack of detail on their severity. A few studies provided detail on symptom severity.^28,36^
• The lack of matched controls for most of the studies means that it was difficult to ascertain which symptoms were actually linked to long COVID and which might be related to ageing or co-morbidities.
• Majority of the studies recruited previously hospitalised patients therefore there is a lack of data on long COVID in patients who had mild-to moderate acute infections self-managed at home.
• Some studies involved patients with suspected COVID-19, in the absence of confirmatory testing, which raises the possibility that some of the symptoms reported might actually be related to other infections.^35,36^
• A few studies obtained their data from social media and internet sources, which may not be entirely reliable or verifiable.^13,21^
• Some studies relied on self-selection, and patient requests for follow-up which might have led to selection bias.^16,18^
• A number of studies included in this review are still undergoing peer review and so care needs to be taken when interpreting or using their findings.^4,13,18,19^

**Figure 2.**
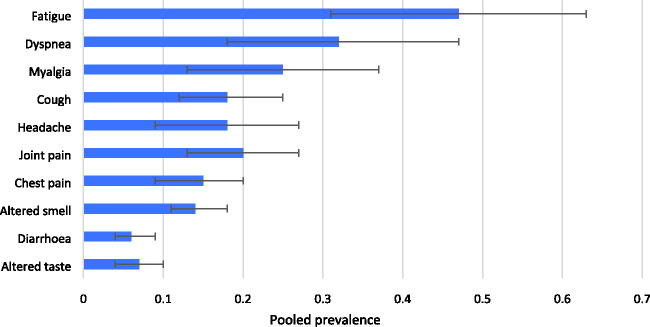
Pooled estimates for the 10 most common symptoms in patients with long COVID-19.

**Figure 3.**
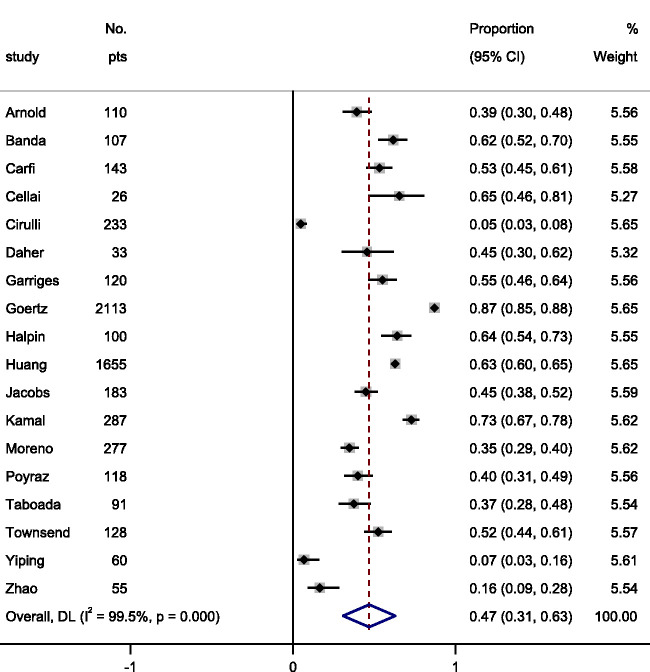
Pooled estimate of the prevalence of fatigue in patients with long COVID-19.

**Figure 4.**
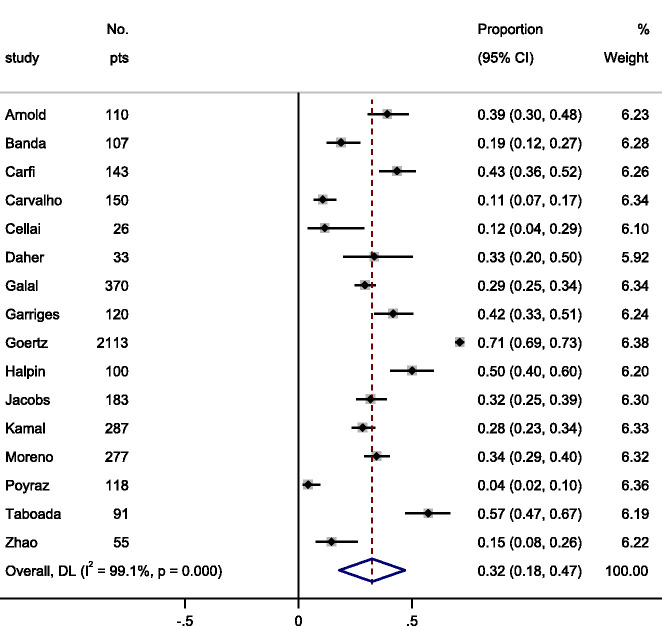
Pooled estimate of the prevalence of dyspnoea in patients with long COVID-19.

**Figure 5.**
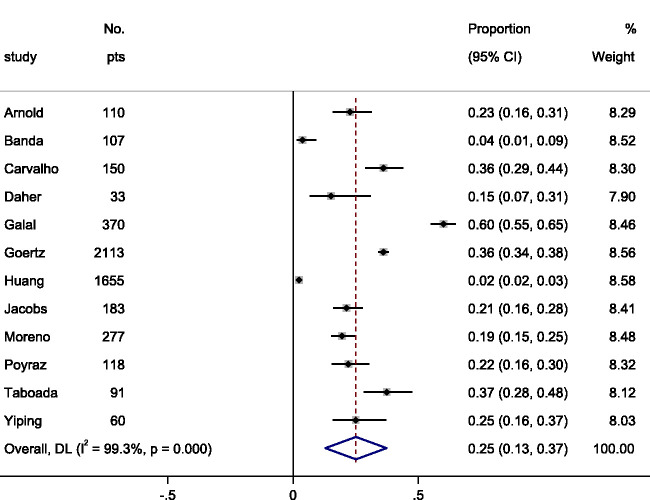
Pooled estimate of the prevalence of muscle pain in patients with long COVID-19.

We pooled prevalence data from the included articles (Figure 2) and the 10 most prevalent reported symptoms were: (i) fatigue 47% (95% CI 31–63); (ii) dyspnoea (shortness of breath) 32% (95% CI 18–47); (iii) myalgia (muscle pain) 25% (95% CI 13–37); (iv) joint pain 20% (95% CI 13–27); (v) headache 18% (95% CI 9–27); (vi) cough 18% (95% CI 12–25); (vii) chest pain 15% (95% CI 9–20); (viii) altered smell 14% (95% CI 11–18); (ix) altered taste 7% (95% CI 4–10); and (x) diarrhoea 6% (95% CI 4–9). Figures 3 to 5 show individual forest plots for fatigue, dyspnoea (shortness of breath) and myalgia (muscle pain) (Figures S1–S7 in Supplement for the others).

Other common symptoms were cognitive impairment ‘brain fog’, amnesia (memory loss), sleep disorder, palpitations (awareness of heartbeat) and sore throat. Less common symptoms such as runny nose, sneezing, hoarseness, ear pain and rare, but important outcomes, including thoughts of self‐harm and suicide, seizures, and bladder incontinence, were only reported by the ongoing symptomatic COVID-19 studies. Conversely, hair loss, hearing loss and tremors were only reported by articles focused on post-COVID-19 syndrome.^20,23,26^ Sicca syndrome, also known as Sjögren syndrome, a condition characterised by dry eyes and dry mouth, was reported by only one study.^13^ Two main symptom clusters of long COVID were identified namely: (i) those comprising exclusively of fatigue, headache and upper respiratory complaints; and (ii) those with multi-system complaints including ongoing fever and gastroenterological symptoms.^4^

#### Long COVID symptoms in relation to severity of acute COVID-19

The presence of more than five symptoms in the first week of acute infection was significantly associated with the development of long COVID irrespective of age or gender.^4^

Although the number of symptoms have been shown to decline from acute COVID-19 to follow-up,^29^ several studies demonstrated that a significant number of patients continue to experience persistent symptoms regardless of the severity of the initial illness.^14–16,18,35^ For instance, a study that grouped patients as having mild, moderate or severe acute COVID-19 based on their need for oxygen supplementation and/or intensive care showed 59% (16/27) of patients with mild disease continued to experience COVID-19 symptoms at 8–12 weeks of follow-up from symptom onset.^35^ In comparison, 75% (49/65) and 89% (16/18) of patients in the moderate and severe groups had ongoing symptoms within the same period.^35^ Breathlessness and fatigue were the most common symptoms regardless of the severity of the acute illness.^35^

In a single-centre study of 143 previously hospitalised patients, 87.4% had at least one symptom (predominantly fatigue and dyspnoea) and 55% reported three or more symptoms at the time of assessment, approximately eight weeks from the onset of the first symptom.^14^

A study of 26 patients with mild COVID-19 reported that 96.2% experienced at least one symptom and 69.2% had four or more symptoms six weeks after symptom onset.^16^ Similarly, another study found that in a sample of 150 patients with mainly mild-to-moderate acute COVID-19 (77.3%, 116/150), at least one persistent symptom was reported at eight weeks in 66% of patients.^15^ Notably, anosmia/ageusia (loss of sense of smell/taste), myalgia (muscle pain) or headache were the symptoms that persisted in patients with mild-to-moderate COVID-19 in this study.^15^ In a sample of patients with mostly mild confirmed infection (96.6%, 225/233), a study found that nearly a quarter still had at least one symptom after 12 weeks.^18^ Finally, a study with a six-month follow-up after symptom onset found that 76% (1265/1733) of previously hospitalised patients reported at least one symptom, and the proportion was higher in women.^23^ Women were also found to have higher odds for fatigue or weakness than men.^23^

### Determinants of the occurrence of persistent symptoms

Older age,^4,15,24^ female gender,^4,24^ hospital admission at symptom onset,^4,15^ initial dyspnoea,^15,18^ chest pain,^18^ abnormal auscultation findings (sounds from the heart, lungs or other organs),^15^ symptom load during the acute phase^4,29^ and co-morbidities^29^ particularly asthma^4^ were found to be significantly associated with an increased risk of developing persistent symptoms. The need for oxygen therapy, pre-existing hypertension and chronic lung conditions were highlighted as the main determinants of long-term symptoms.^19^

Older age, self-reported health status before the onset of symptoms, pre-existing co-morbidities and the number of symptoms during acute infection were found to significantly predict the number of symptoms patients with long COVID may experience at follow-up.^21^ Although Moreno-Pérez et al. found a significant association between anosmia-dysgeusia and younger age (<65 years) at 10–14 weeks,^27^ this was not statistically significant in the analysis done by another study at 8 weeks.^32^

### Lived experience

The mid- and long-term effects and impact of illness due to COVID-19 is yet to be fully understood. However, there is evidence that the impact of acute COVID-19 on patients, regardless of severity, extends beyond hospitalisation in severe cases, to ongoing impaired quality of life, mental health and employment issues.^14,15,17,24^ People living with long COVID have indicated that they are suffering with a range of symptoms, feel ‘abandoned’ and ‘dismissed’ by healthcare providers and receive limited or conflicting advice.^38^ More than one-third (48/130) of the patients in a study reported they still felt ill or in a worse clinical condition at eight weeks than at the onset of COVID-19.^15^

### Quality of life

The generic EuroQol Five Dimension (EQ-5D) index score, EuroQol Visual Analog Scale (EQ-VAS),^3,14,23,27,30^ RAND Short Form-36 questionnaire (SF-36)^35^ and the PROMIS® Global Health instrument^33^ were used to assess the quality of life of patients with long COVID. Existing evidence suggests that people with long COVID experience significant reductions in quality of life.

#### Quality of life at 4–12 weeks

In a study of previously hospitalised patients with COVID-19 in the United States, scores on the PROMIS® Global Health-10 instrument indicated worse general health after their acute illness compared to baseline.^33^ The patients’ summary *t* scores in both the physical health and mental health domains were slightly above the United States mean at baseline. However, both scores were significantly lower and patients reported a reduced ability to carry out social activities 4–6 weeks after hospitalisation.^33^

Overall EQ-5D index score was 0.86 (standard deviation 0.20) and EQ-VAS was 70.3% (standard deviation 21.5) at approximately eight weeks in a cohort of patients who had severe acute COVID-19.^3^ Another study stated that previously hospitalised patients had an average EQ-VAS score of 63%, and based on their data, 44.1% of the patients experienced a reduction in quality of life (defined as a 10-point difference in health status) before COVID-19 versus eight weeks of follow-up.^14^ However, these findings are difficult to interpret without data from age-/gender-matched controls without COVID-19.

Scores obtained from the administration of the SF-36 at 8–12 weeks to patients with mild, moderate and severe acute COVID-19 showed an impairment in reported health status across all domains compared with age-matched population norms.^35^ Physical scores were significantly lower in the severe group in comparison to patients in the mild-to-moderate groups.^35^

#### Quality of life at 12 weeks or more

A comparison of EQ-VAS scores showed a significant difference in overall quality of life for patients with ongoing symptoms and those who reported no symptoms after their acute infection at 10–14 weeks (43.2% vs. 66.9%, p = 0.0001).^27^ In a study with a six-month follow-up of previously hospitalised patients, an overall EQ-VAS score of 80% was reported indicating persistent reductions in quality of life.^23^

At six months of follow-up, in a study of previously hospitalised patients with COVID-19–related acute respiratory distress syndrome (ARDS), 67% (61/91) had a decrease in their quality of life.^30^ Comparison of EQ-5D index scores before acute infection and six months after showed a significant difference in quality of life (*before* = 0.965, *after* = 0.705, p < 0.001). Their EQ-VAS scores were also significantly different (*before* = 87.6%, *after* = 66.4%, p < 0.001).^30^ Similarly, there was a significant impairment in functional status among the patients with only 30.8% reporting no limitations in their everyday life (based on the Post-COVID-19 Functional Status scale).^30^ In a study that included individuals who regularly engaged in sports before hospitalisation for COVID-19 (details of the sporting activities were not given), 72% (28/39) were able to resume physical activity after three months, and nearly half of those were only able to do so at lower intensity.^20^

### Impact on mental health

At eight weeks after acute infection, nearly half of all patients (238/488) surveyed in a study were emotionally affected by their experiences of long COVID with 28 requiring further mental health care.^17^ At six months of follow-up after the onset of symptoms, another study found that 23% of previously hospitalised patients suffered from anxiety or depression with women having higher odds than men.^23^

However, a study that utilised the Warwick-Edinburgh Mental Wellbeing Scales reported its scores were comparable with published population norms, and there were no significant differences between patients with mild, moderate or severe infection.^35^ According to data from Patient Health Questionnaire 9 (PHQ-9) and Generalized Anxiety Disorder 7 (GAD-7) questionnaires administered to a group of patients with severe acute COVID-19 approximately eight weeks after hospital discharge, patients mostly suffered from mild depression and anxiety.^3^

A study conducted in Turkey focused on the mental health of patients previously treated at a tertiary hospital at eight weeks of follow-up. Data collected using the Impact of Events Scale-Revised (IES-R) showed a quarter of the patients (72/284) had moderate-to-severe post-traumatic stress disorder (PTSD) symptoms while 18.3% (52/284) had mild PTSD symptoms.^28^ Over 40% reported co-morbid depression. Based on responses for the Mini-International Neuropsychiatric Interview suicidality scale, 7.4% (21/284) patients had a positive response to one or more items. Of these, six had a ‘moderate’ current risk of suicide, based on their Mini-International Neuropsychiatric Interview combined score.^28^ In addition, the study found that the occurrence of PTSD was significantly higher and more severe in women. Patients with severe acute COVID-19 had a significantly higher occurrence of PTSD symptoms and those with a higher mean acute symptom burden where more likely to exhibit PTSD symptoms.^28^ However, a significant proportion of patients with moderate-to-severe PTSD symptoms had a past psychiatric diagnosis.

Inadequate social support was linked to the occurrence and severity of PSTD symptoms.^28^ Social stigmatisation and discrimination appeared to influence the severity of PTSD symptoms as the study found that patients who felt stigmatised were more likely to experience moderate-to-severe PTSD symptoms.^28^ COVID-19–related stigmatisation has also been linked to a sense of hopelessness in patients.^38,39^

### Impact on employment

A study found that among 195 patients who were employed before hospitalisation, 40% were unable to return to work within eight weeks of discharge due to ongoing health problems or job loss.^17^ Of those who returned to work, a quarter needed to reduce their working hours or alter their duties for health reasons.^17^ Another study reported nearly 70% (38/56) of previously hospitalised patients were unable to return to work at three months after hospitalisation.^20^

### Risk of readmission

Ongoing fever and skipped meals were reported to be strong predictors of a subsequent hospital visit.^4^ A few studies have reported incidences of readmission of previously hospitalised patients with long COVID ranging from 1.4% to 15%.^17,23,33^ The study by Chopra et al. found that 15% (189) of previously hospitalised patients were symptomatic enough to require readmission within eight weeks of discharge.^17^

### Complications associated with long COVID-19

COVID-19 is a multi-systemic disease, which may occur with complications at presentation or developing during the acute phase of illness. These complications may be respiratory,^40^ cardiovascular,^41–45^ renal,^46–48^ gastrohepatic,^49–53^ thromboembolic,^54–58^ neurological,^59–61^ cerebrovascular^62–64^ and autoimmune^13,65^ among others.

Beyond persistent symptoms, patients with long COVID may have clinical complications related to the disease.^39^ The epidemiology and pathophysiology of complications in long COVID are presently not well understood. While some studies have described some initial findings which are presented here, there is an urgent need for research into the underlying mechanisms (Box 2).^66,67^

**Box 2.** Priority areas and considerations for future research.

• Treatment options are currently limited as there is insufficient understanding of the mechanisms that underpin long COVID. Longer-term longitudinal observational studies are needed to fully understand the pathophysiology of the symptoms and complications associated with long COVID-19, its clinical course, symptom clusters and syndromes. This evidence will be crucial to understand the natural history of long COVID and the types of interventions that may be required. Qualitative research into the lived experiences of patients could provide the insight required for the planning of effective care pathways and lead to improved clinical outcomes. Clinical trials are urgently needed to evaluate interventions for long COVID that address the wide range of symptoms and complications identified in this review.
• Racial differences in the incidence of acute COVID-19 infections have been well documented. However, such differences have not been well researched in patients with long COVID and need further exploration.
• Most studies have focused on hospitalised patients and there is an urgent need for studies to investigate long COVID in non-hospitalised COVID-19 patients who have been underrepresented in the current research literature.

### Cardiovascular abnormalities

Evidence of myocarditis or prior myocardial injury by cardiac magnetic resonance imaging was found in 12/26 (46%) college athletes 12–53 days after their acute COVID-19 infection despite the fact that none were hospitalised; less than half had mild symptoms and the rest asymptomatic.^68^ A study of 100 patients showed that 78% had abnormal findings based on cardiac magnetic resonance imaging results 2–3 months after the onset of COVID-19 and 60% had evidence of myocardial inflammation independent of the severity and overall course of their acute illness.^69^ As with most of the literature available on long COVID, the sample selection was not random and may be biased. However, the possibility of cardiovascular abnormalities occurring in patients with long COVID was supported by another study which reported that up to 40% of COVID-19 patients presented with pericarditis or myocarditis > 70 days after infection.^70^

### Pulmonary abnormalities

#### Lung function tests

A study conducted lung function tests in a sample of 57 patients 30 days after discharge for acute COVID-19 and reported a decrease in lung diffusion capacity for carbon monoxide (DLCO) in 53% and diminished respiratory muscle strength in 49% of patients.^71^ In another study, lung function abnormalities were detected in approximately a quarter of patients (14/55) at three months after hospital discharge.^34^ The commonest lung function abnormality (16.36%) was DLCO. A higher level of D-dimer at admission was significantly associated with DLCO% < 80% suggesting that D-dimer might be a potential biomarker for the prediction of DLCO decline patients with COVID-19.^34^ Of the patients with lung function abnormalities, 12 also had radiological changes, including evidence of lung fibrosis.^34^ At six months of follow-up, Huang et al. also found lung diffusion impairment among 34% (114/334) of patients previously hospitalised for acute COVID-19.^23^

#### Chest computed tomography scans

Varying degrees of radiological abnormalities in the chest computed tomography scans of 71% (39/55) of patients who were previously admitted for COVID-19 were discovered by another study at approximately three months after discharge.^34^ One to three lung segments were involved in about half of the patients with radiological abnormalities. Thirteen patients (23.64%) showed bilateral involvement and 15 (27.27%) had evidence of fibrosis (interstitial thickening).^34^ Persistent symptoms were also reported by 64% of the patients.^34^ Ground glass opacity was the most common high-resolution computed tomography pattern observed in the study by Huang et al. at six months after discharge.^23^

### Neurological abnormalities

The occurrence of encephalitis, seizures and other conditions such as major mood swings and cognitive impairment (brain fog) have been reported in patients up to two to three months after the onset of acute illness.^72^ Magnetic resonance imaging scanning (diffusion tensor imaging and three-dimensional T1-weighted imaging) of previously hospitalised patients with COVID-19 suggested possible disruption to micro-structural and functional brain integrity at three months of follow-up,^26^ thus signifying the neuro-invasive capabilities of the SARS-CoV-2 virus and the potential for long-term consequences of the infection.

### Renal complications

In one study, approximately one-third of previously hospitalised patients, who had acute kidney injury during the acute phase of COVID-19, did not fully regain renal function at discharge or post-hospitalisation follow-up.^73^

### Endocrine disorders

Two studies reported newly diagnosed diabetes mellitus in patients after hospitalisation.^25,74^ However, more research is required to fully understand the aetiology.

### Comparison to other coronaviruses

Although the clinical manifestations of COVID-19 are distinct, the persistence of dyspnoea and fatigue were similarly reported for the severe acute respiratory syndrome (SARS) and the Middle East Respiratory Syndrome (MERS) coronavirus infections.^22,24^ The findings on pulmonary abnormalities in patients with long COVID are similar to those from a study of patients, who recovered from SARS, but still had abnormal computed tomography findings and DLCO anomalies after a year.^75^ A meta-analysis of 28 follow-up studies found that six months after hospital discharge, approximately 25% of patients hospitalised with SARS and MERS had reduced lung function and exercise capacity.^22,76^

In the longer term, PTSD, depression and anxiety, and reduced quality of life were observed at one year after infection with SARS and MERS.^76^ In addition, a study found that up to 40% of patients who had SARS continued to experience fatigue and psychiatric illnesses for nearly 3.5 years after the acute infection.^77^ These findings are similar to those from a six-month follow-up study of previously hospitalised patients with COVID-19, which showed that patients mainly struggled with fatigue or muscle weakness, sleep difficulties, and anxiety or depression.^23^ This suggests that in the longer term, patients with long COVID may also experience a similar disease trajectory to that of patients who had SARS or MERS.^22^

### Management of long COVID

Treatment options are currently limited as there is insufficient understanding of the mechanisms that underpin long COVID (Box 2). While there are still uncertainties about the optimal management of patients with long COVID, a number of countries have produced clinical guidelines to assist clinicians.^11,78^ Patients may require multidisciplinary care involving the long-term monitoring of ongoing symptoms, to identify potential complications for clinical intervention and the need for physical rehabilitation, mental health and social services support (Figure 1).

### Aspects of management

#### Physical rehabilitation

Patients with severe acute COVID-19 who are managed in intensive care units may develop muscle weakness, deconditioning, myopathies (muscle disease) and neuropathies (nerves damage or dysfunction), which are the physical domains of post-intensive care syndrome.^79^ It is recommended that appropriate rehabilitation to prevent this syndrome should start in intensive care units as soon as sedation and clinical stability permit.^79^ Pulmonary rehabilitation may help improve patients’ breathing, exercise capacity, muscle strength, quality of life and functional outcome.^80^ Early mobilisation would help to improve functional, cognitive and respiratory conditions in these patients and may shorten hospital stay.^79^

Non-hospitalised patients with long COVID may also require physical rehabilitation, especially those with cardiopulmonary problems who may need significant rehabilitation, in order to improve their ability to engage in activities of daily living. However, identifying this group of patients may be challenging due to under-recognition and under-investigation of symptoms. There is also a risk that non-hospitalised patients with long COVID with mild-to-moderate symptoms, who are likely to represent a significant proportion of long COVID sufferers, may not be prioritised for follow-up care.^38^

#### Management of pre-existing co-morbidities

A significant proportion of patients who experience severe acute COVID-19 have underlying co-morbidities. It is therefore essential that these are adequately managed in order to avoid clinical deterioration and the need for readmission in these patients.^81^

#### Mental health support

Psychological and mental health issues such as anxiety, depression, PTSD and suicidal ideation have been discussed earlier as some of the long-term consequences of long COVID.^16,19,22,25,26,28^ There is a need to ensure that appropriate mental health support is available and accessible to those patients who require such services. Patients may be screened as part of their follow-up care and those identified as requiring extra support referred for specialist management. However, care should be taken not to pathologise patients as physical manifestations of COVID-19 may distort responses to assessment tools.^81^

#### Social services support

Due to persistent symptoms, a significant number of patients with long COVID are unable to return to work and may require long-term governmental financial support.^17,20^ Some patients may be unable to cope with day-to-day living especially if they also suffer significant social isolation and or stigmatisation.^38,39^ These groups of patients would benefit from social services support.

### Strategies to facilitate the management of patients

#### A role for patient-reported outcomes

Patient-reported outcomes may be used for long-term follow-up care of patients with long COVID to monitor their symptoms and assess the impact on quality of life. The collection and use of patient-reported outcomes can help identify patients with ongoing symptoms, especially those who were not previously hospitalised and so not receiving formal follow-up. There is evidence that patient-reported outcomes are capable of detecting adverse events in patients even before clinical parameters.^82^ Patient-reported outcome data may alert clinicians to the development of potentially life-threatening complications in patients with long COVID.^83^ As shown by a number of studies discussed in this review, patient-reported outcome data may also indicate which patients are struggling to cope physically and mentally with their condition.^14,23,30,35^ In research settings, they can also provide valuable information on the effectiveness, safety and tolerability of drug interventions.^84^ The International Consortium for Health Outcomes Measurement initiative has developed a core outcome set for COVID-19 studies.^85^ Adoption of such standards would enable the collection of globally comparative data.

#### Harnessing the capabilities of digital technologies

Digital technologies are currently being used for the public health response to the COVID-19 pandemic through population surveillance, case identification, contact tracing and evaluation of interventions.^86^ A study found up to 30.5% (382) of previously hospitalised patients required follow-up with a primary care physician. Of these, 42% was via videoconferencing.^17^ Where possible, videoconferencing could be used for follow-up of patients with long COVID. This would also reduce the need for in-person contact and the risk of reinfection while the pandemic continues.

Moving forward, a digital therapeutics approach could be implemented where non-pharmacological interventions such as rehabilitative breathing exercises can be delivered via a digital platform according to patients’ presentations where feasible. This may ensure that a greater number of patients are cared for than would be possible with in-person care alone. Dedicated in-person COVID-19 rehabilitation services would require a substantial amount of resources^87^ and tele-rehabilitation may be potentially cost-effective in the long-term.

Advances in digital technology have facilitated the collection of electronic patient-reported outcome data. Electronic patient-reported outcomes and other measures such as temperature, oxygen saturation and blood pressure (measurable by wearable devices) may be collected remotely for sharing with clinical teams. Such data can also be analysed using machine learning and artificial intelligence to monitor and identify at-risk patients for early clinical intervention and rehabilitation.^88^

## Conclusion

The wide range of potential symptoms and complications patients with long COVID may experience highlights the need for a deeper understanding of the clinical course of the condition. There is an urgent need for better, more integrated care models to support and manage patients with long COVID-19 in order to improve clinical outcomes. Resilient healthcare systems are required to ensure efficient and effective responses to future health challenges.

## Supplemental Material

sj-pdf-1-jrs-10.1177_01410768211032850 - Supplemental material for Symptoms, complications and management of long COVID: a review Click here for additional data file. Supplemental material, sj-pdf-1-jrs-10.1177_01410768211032850 for Symptoms, complications and management of long COVID: a review by Olalekan Lee Aiyegbusi, Sarah E Hughes, Grace Turner, Samantha Cruz Rivera, Christel McMullan, Joht Singh Chandan, Shamil Haroon, Gary Price, Elin Haf Davies, Krishnarajah Nirantharakumar, Elizabeth Sapey, Melanie J Calvert and on behalf of the TLC Study Group in Journal of the Royal Society of Medicine
