# Supplementary material for: Symptoms, complications and management of long COVID: a review
Source: J R Soc Med. 2021 Jul 15;114(9):428–42. doi: 10.1177/01410768211032850 (PMC8450986; doi:10.1177/01410768211032850)
Supplement: sj-pdf-1-jrs-10.1177_01410768211032850 - Supplemental material for Symptoms, complications and management of long COVID: a review [file sj-pdf-1-jrs-10.1177_01410768211032850.pdf]

Supplement

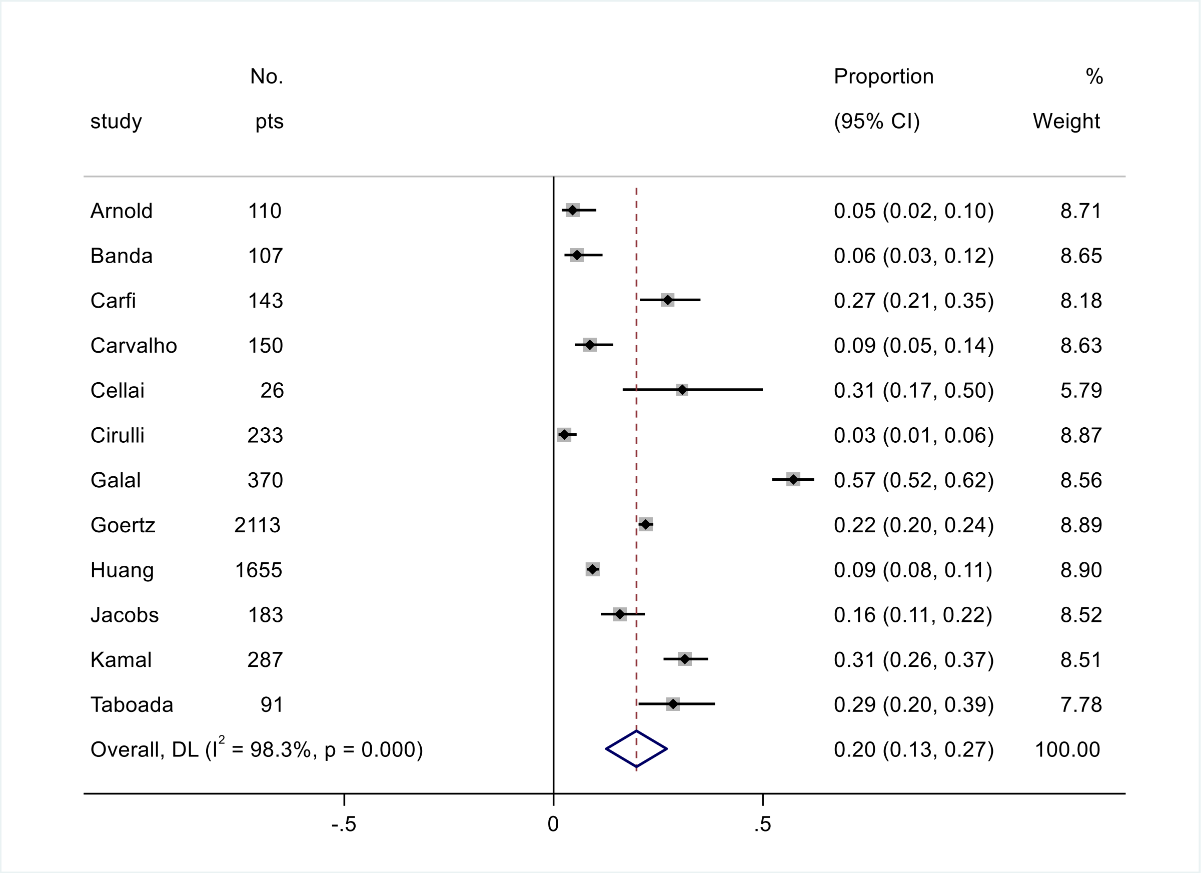

Figure S1. Pooled estimate of the prevalence of joint pain in patients with long COVID-19

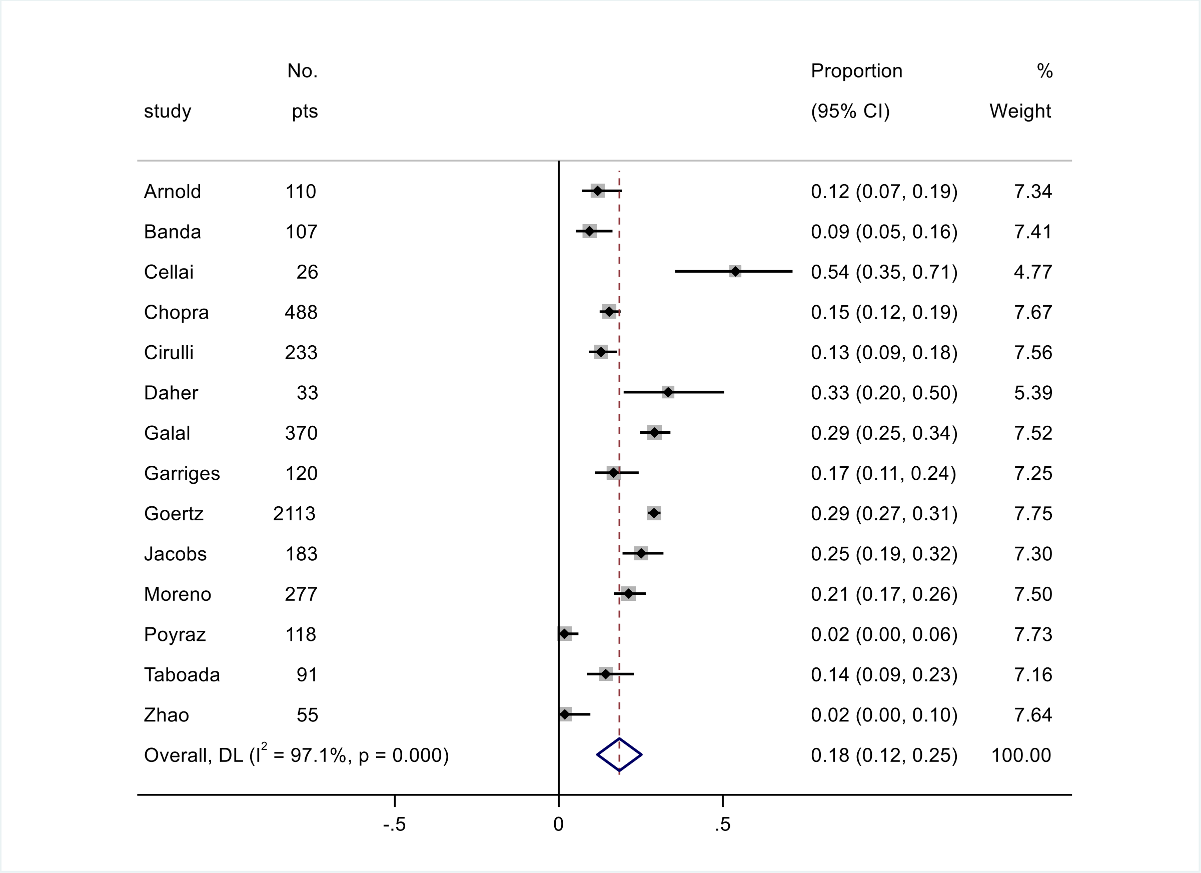

Figure S2. Pooled estimate of the prevalence of cough in patients with long COVID-19

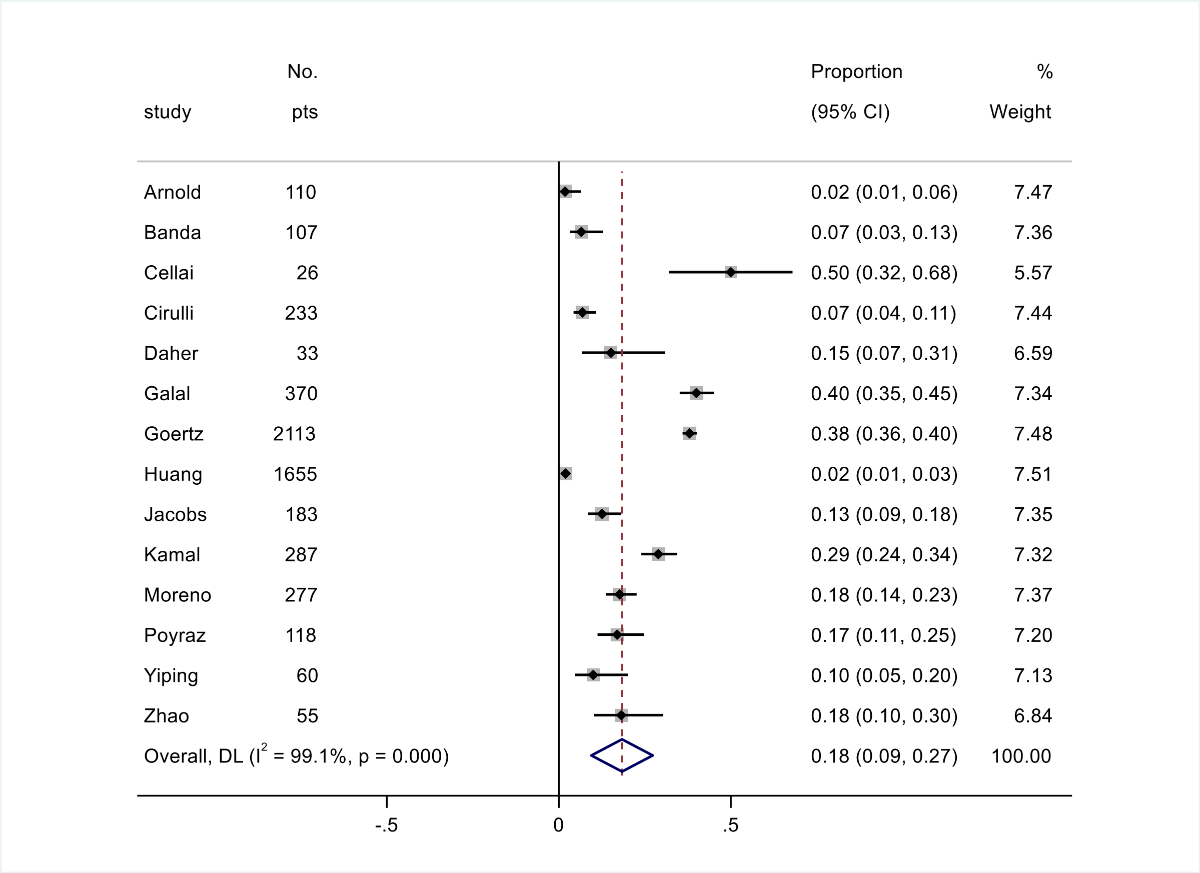

Figure S3. Pooled estimate of the prevalence of headache in patients with long COVID-19

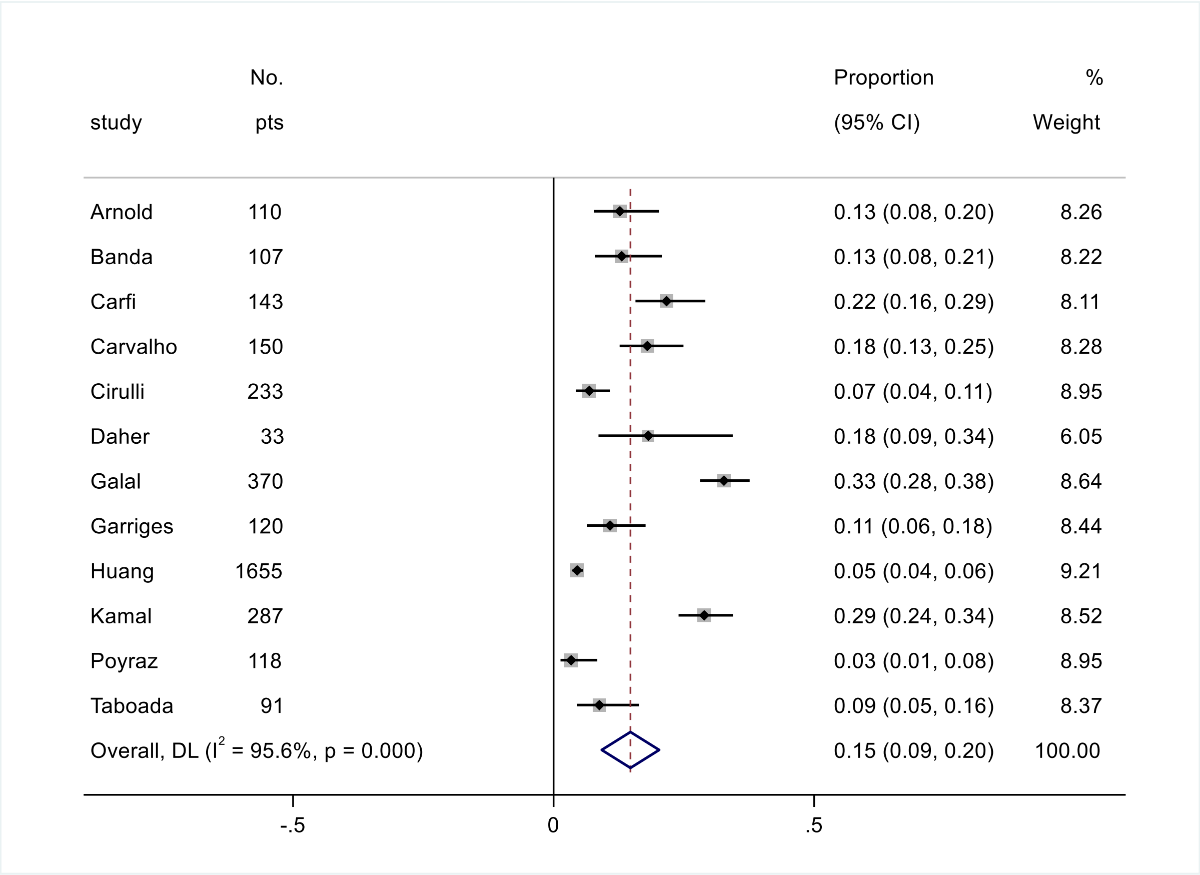

Figure S4. Pooled estimate of the prevalence of chest pain in patients with long COVID-19

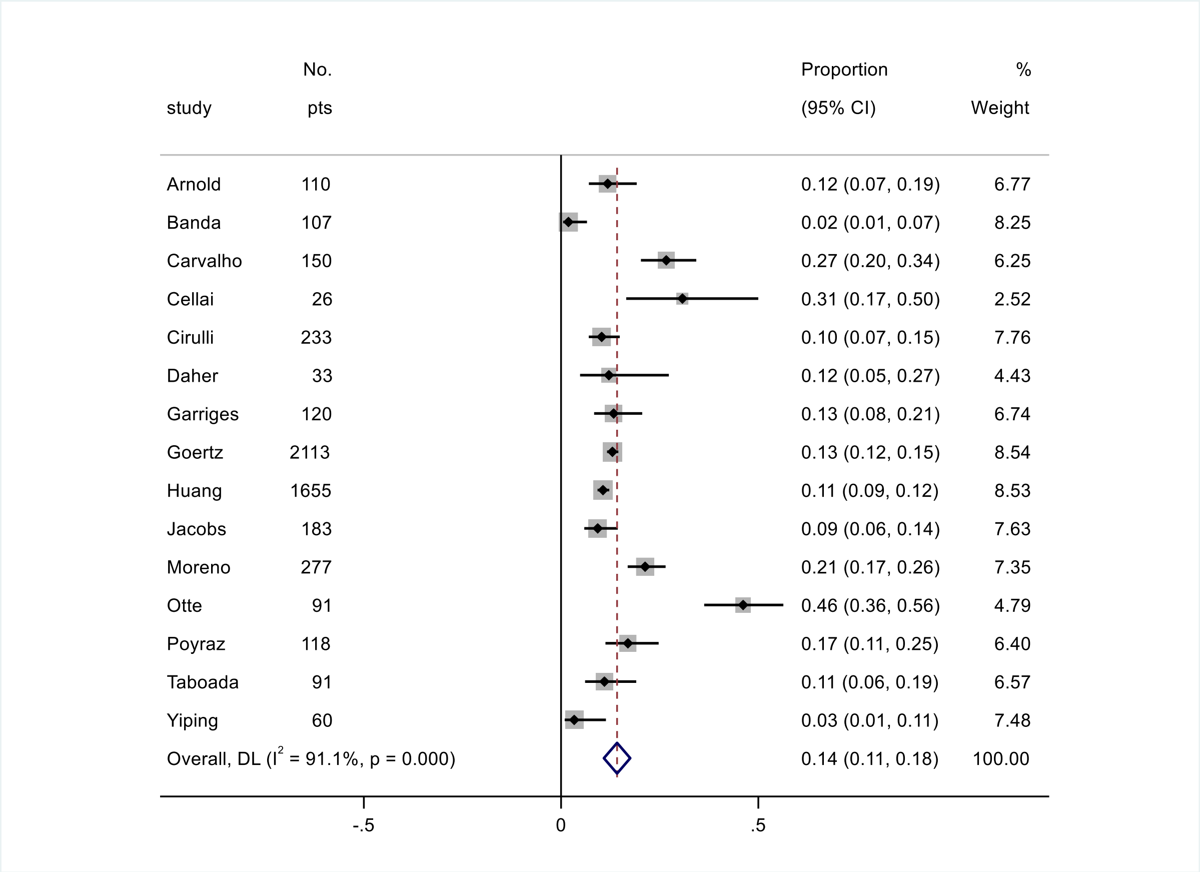

Figure S5. Pooled estimate of the prevalence of altered smell in patients with long COVID-19

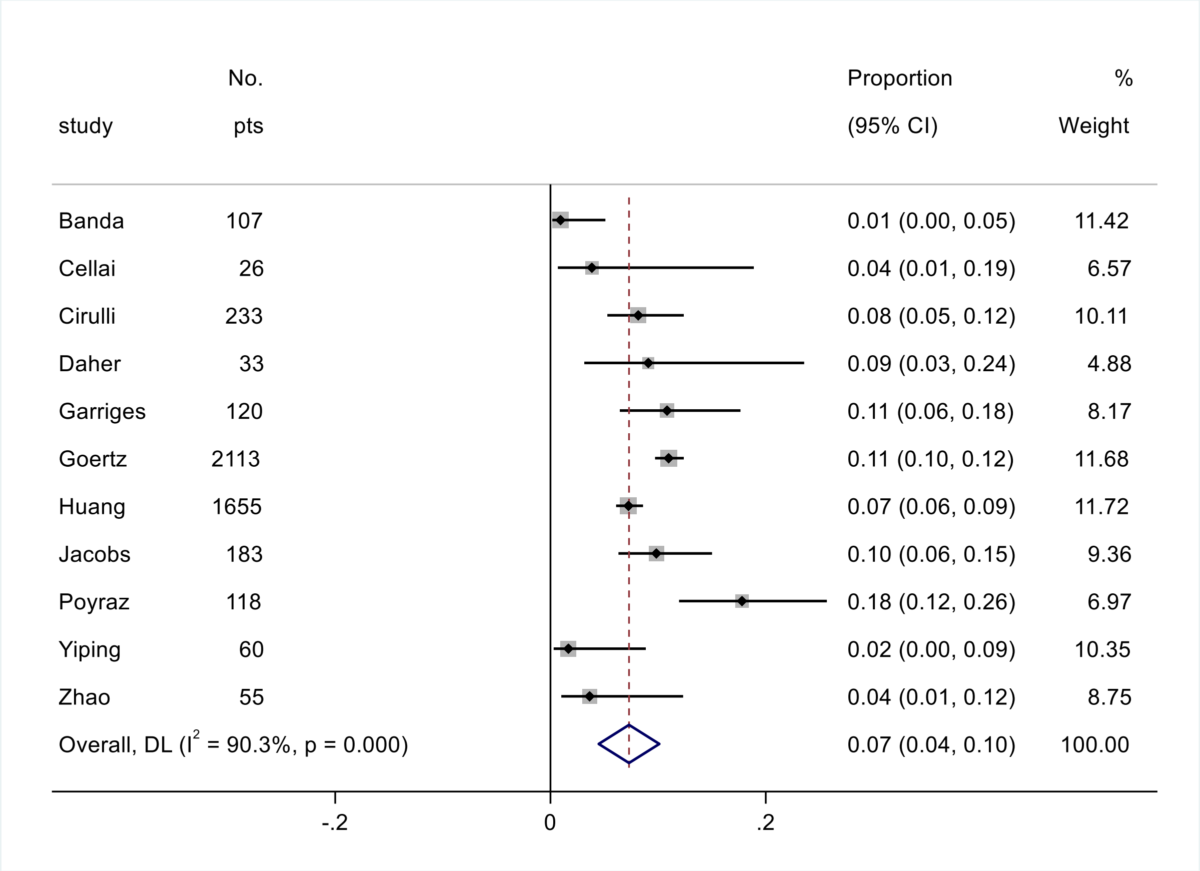

Figure S6. Pooled estimate of the prevalence of altered taste in patients with long COVID-19

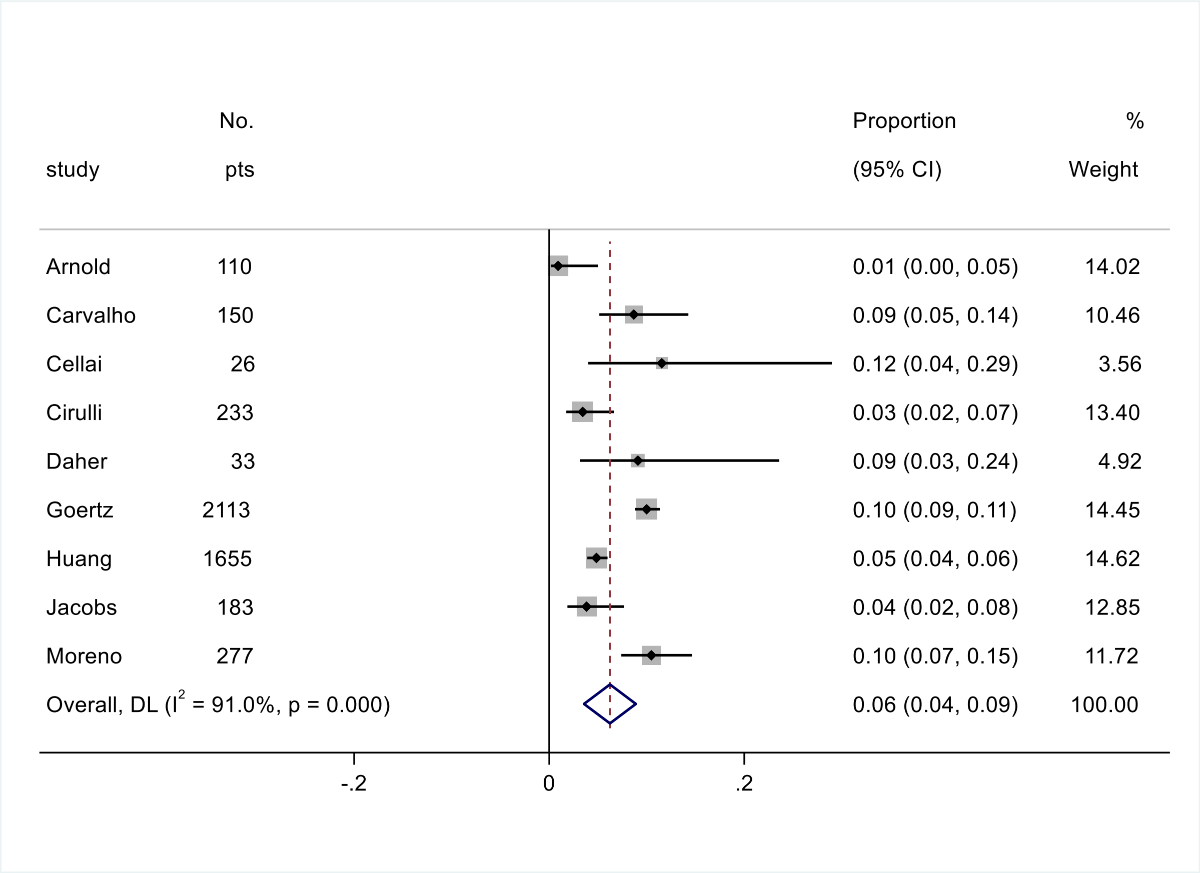

Figure S7. Pooled estimate of the prevalence of diarrhoea in patients with long COVID-19
